# Association between metabolic dysfunction-associated steatotic liver disease and kidney stone risk in individuals with metabolic dysfunction: evidence from cross-sectional and cohort analyses

**DOI:** 10.3389/fendo.2026.1752891

**Published:** 2026-02-02

**Authors:** Yushuang Wei, Lingyu Ye, Mingli Li, Boteng Yan, Yining Lin, Sihua Lai, Zengnan Mo, Chaoyan Tang

**Affiliations:** 1 School of Public Health, Guangxi Medical University, Nanning, Guangxi, China; 2 Institute of Urology and Nephrology, First Affiliated Hospital of Guangxi Medical University, Guangxi Medical University, Nanning, Guangxi, China; 3 Center for Genomic and Personalized Medicine, Guangxi key Laboratory for Genomic and Personalized Medicine, Guangxi Collaborative Innovation Center for Genomic and Personalized Medicine, Guangxi Medical University, Nanning, Guangxi, China; 4 School of Basic Medical Sciences, Guangxi Medical University, Nanning, Guangxi, China; 5 Department of Endocrinology, The First People’s Hospital of Yulin, Yulin, Guangxi, China

**Keywords:** CKM syndrome, kidney stones, metabolic dysfunction, metabolic dysfunction-associated steatotic liver disease, METS-IR score

## Abstract

**Introduction:**

Kidney stones are a common disorder with increasing global prevalence. Metabolic dysfunction-associated steatotic liver disease (MASLD), a systemic metabolic condition, has been suggested to be linked with kidney stones, but existing evidence is inconsistent. This study aimed to clarify the association between MASLD and kidney stones risk using both cross-sectional and cohort analyses.

**Methods:**

A total of 1,875 participants from a cross-sectional study and 1,903 from a community-based cohort were analyzed. Logistic regression was used in the cross-sectional study, while incidence rates, Kaplan–Meier curves, log-rank tests, and Cox models estimated risk in the cohort. Subgroup and mediation analyses were performed, with METS-IR, WBC, and eGFR examined as mediators.

**Results:**

In the cohort study, there were 94 incident kidney stone cases identified during a median follow-up of 34.62 months, with an incidence rate of 17.6 per 1,000 person-years. In the cross-sectional analysis, MASLD was positively associated with kidney stones, with an odds ratio (OR) of 1.466 (95% CI: 1.059–2.028) after adjustment for potential confounders. Kaplan–Meier analysis revealed significant differences in cumulative incidence between MASLD and non-MASLD groups (log-rank P < 0.001). Cox regression confirmed MASLD as an independent risk factor for kidney stones (HR = 2.04, 95% CI: 1.29–3.23). Subgroup analyses showed consistent associations in metabolically high-risk individuals. Mediation analyses further highlighted METS-IR as a key mediator linking MASLD to kidney stone formation.

**Conclusions:**

MASLD was independently associated with increased kidney stone risk, particularly in metabolically high-risk individuals. METS-IR mediated this relationship, underscoring the critical role of insulin resistance.

## 1 Introduction

Kidney stones represent a prevalent urological condition worldwide, yet their etiology remains incompletely elucidated. The disease is characterized by a substantial risk of recurrence, rendering it a chronic relapsing disorder that places a persistent burden on patients and health systems alike (1). The prevalence has been estimated at 4%–6% in China, with a steady upward trend documented over the past three decades (2). Calcium oxalate stones constitute the predominant subtype (3). While aberrant urinary composition has long been recognized as a proximate cause, accumulating evidence indicates that systemic metabolic disturbances also play a critical role in stone formation (4). In particular, obesity, insulin resistance (IR), diabetes, and metabolic syndrome have each been robustly linked with elevated stone risk (5, 6). With the global burden of urolithiasis continuing to rise, the identification of metabolic pathways underlying stone formation is essential for advancing risk stratification and informing preventive strategies at both clinical and population levels.

Metabolic dysfunction-associated steatotic liver disease (MASLD), also known as metabolic dysfunction–associated steatotic liver disease, has emerged as a major chronic condition affecting multiple organ systems. It is estimated to affect nearly 38% of the global adult population, thereby posing a significant challenge to public health (7). Accumulating evidence indicates that MASLD exerts detrimental effects not only on hepatic health but also on a wide range of extrahepatic outcomes, including cardiovascular disease, extrahepatic malignancies, and kidney stones (8).

MASLD and kidney stone disease share several upstream metabolic risk factors, including obesity, type 2 diabetes or deglycation, IR, and metabolic syndrome (9, 10). These metabolic abnormalities are well-recognized risk factors for kidney stone formation and also represent key features of the metabolic milieu underlying MASLD. IR commonly accompanies MASLD and has been reported to be associated with alterations in renal uric acid handling and purine metabolism (11, 12). Such metabolic perturbations may partly contribute to an increased susceptibility to kidney stone formation; however, the precise biological pathways linking MASLD-related IR to nephrolithiasis remain to be clarified.

In recent years, increasing attention has been directed toward the potential association between MASLD and kidney stones, however, the available evidence remains inconsistent. In a cross-sectional study, MASLD was independently associated with an increased risk of kidney stones, and the fibrosis-4 index was shown to further stratify this risk (13). A CT-based investigation in China corroborated this association, demonstrating that MASLD significantly elevated the likelihood of kidney stones occurrence (14). Furthermore, a study incorporating both cross-sectional and Mendelian randomization analyses provided evidence for a causal relationship between MAFLD and urolithiasis risk (15). In contrast, findings from a meta-analysis of prospective cohort studies indicated no significant association between MAFLD and incident kidney stones (16). These discrepancies may be attributable to heterogeneity in disease definitions and diagnostic criteria, differences in study design, variations in metabolic characteristics of the study populations, as well as differences in sample size. Therefore, systematic analyses based on real-world data are warranted to further investigate the potential association between MASLD and the risk of kidney stones.

Accordingly, the present study was designed to first examine the association between MASLD and kidney stones through a cross-sectional analysis. This relationship was then further evaluated in a prospective cohort to test potential causal links. In addition, mediation analyses were conducted to explore underlying mechanisms that may account for the observed association. Collectively, these analyses were intended to provide scientific evidence to inform the early prevention and management of kidney stones.

## 2 Methods

### 2.1 Study design and participants

**Figure 1:**
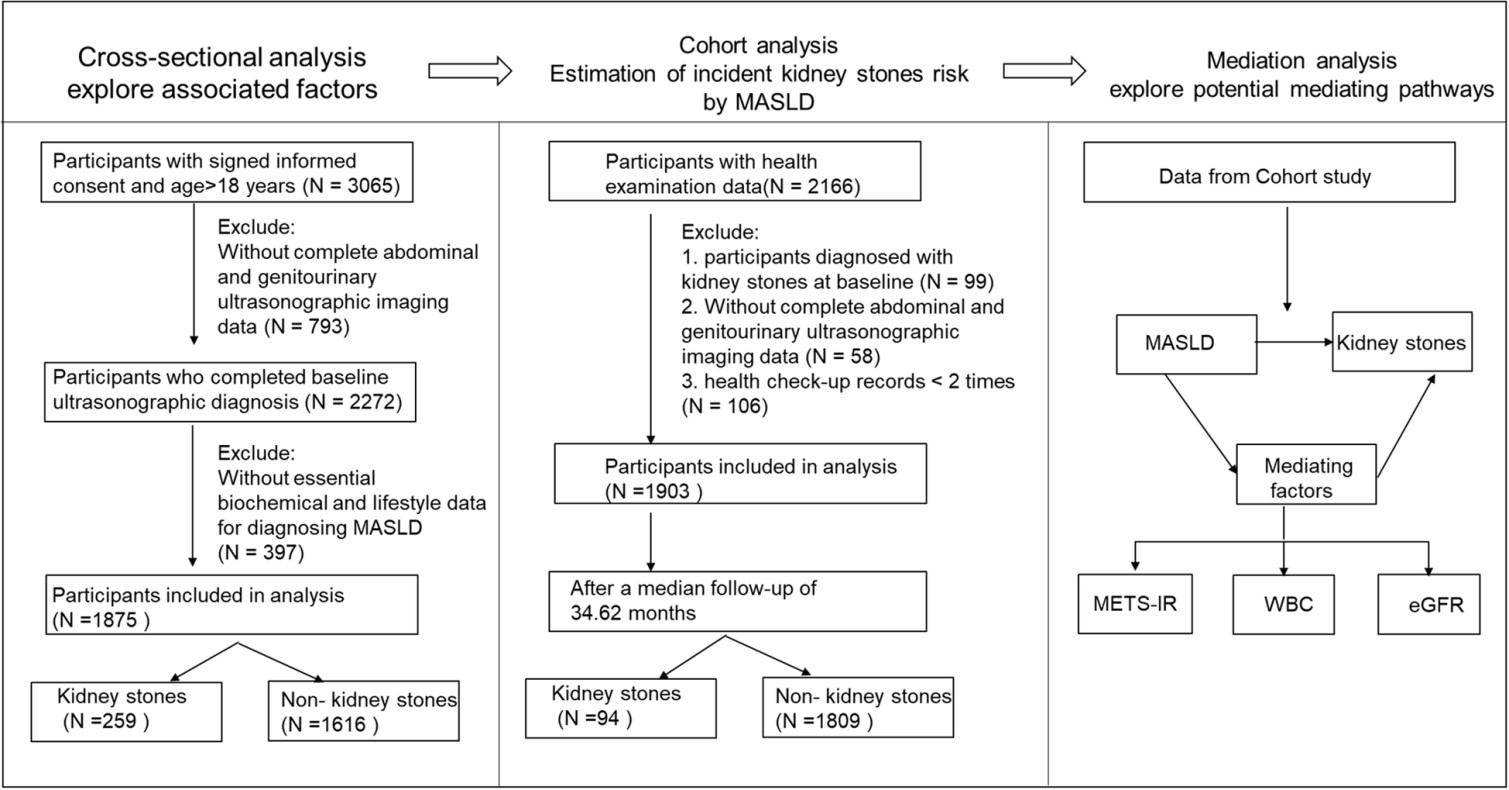
Flowchart of study design and analyses investigating the association between MASLD and kidney stones. MASLD, metabolic dysfunction-associated steatotic liver disease; METS-IR, metabolic score for insulin resistance; white blood cell count (WBC), and estimated glomerular filtration rate (eGFR).

This study consisted of a cross-sectional study and a cohort study. The cross-sectional study included individuals who underwent health examinations at the Health Management Center of the First People’s Hospital of Yulin between January and June 2024, yielding 3065 participants. The cohort study was based on adults who participated in annual health examinations provided by the National Basic Public Health Service Program at the Wuliqiao Community Health Service Center of the First People’s Hospital of Yulin from 2022 to 2024, with 2272 participants enrolled. For the cross-sectional analysis, inclusion criteria were: age ≥18 years, provision of written informed consent, completion of the baseline examination, and availability of abdominal and urinary system ultrasonography data. Exclusion criteria were: missing complete abdominal and urinary system ultrasonography data (n=793) or missing biochemical indicators or lifestyle information required for MASLD classification (n=397). A total of 1,875 participants were included in the final analysis, including 259 with kidney stones and 1,616 without kidney stones. For the prospective cohort analysis, inclusion criteria were: age ≥18 years, provision of written informed consent, availability of follow-up health examination records, completion of baseline abdominal and urinary system ultrasonography, and at least ≥2 health examination visits to support outcome ascertainment during follow-up. Exclusion criteria were: kidney stones diagnosed at baseline (n=99), missing complete abdominal and urinary system ultrasonography data (n=58), or fewer than two health examination records (n=106). Ultimately, 1,903 participants were included, with a median follow-up of 34.62 months (interquartile range: 28.28–38.06 months); during follow-up, 94 incident kidney stone cases occurred, while 1,809 participants remained free of kidney stones. (**Figure 1**).

This study was approved by the Ethics and Human Subjects Committee of Guangxi Medical University, China (No. 2022-0193), and the Ethics and Human Subjects Committee of the First People’s Hospital of Yulin City, China. Written informed consent was obtained from all participants at the time of enrollment.

### 2.2 Demographic data collection

Information on sociodemographic characteristics, lifestyle factors, and medical history (disease and medication use) was obtained through standardized face-to-face questionnaires administered by trained investigators. Smoking status was classified as never, former, or current. Alcohol consumption was stratified into three categories: never, occasional, and daily. Physical activity was likewise categorized as never, occasional, or daily. Anthropometric measurements were performed according to a standardized protocol. Height and weight were automatically measured using an Omron HNH-219 device, and body mass index (BMI) was calculated as weight (kg)/height² (m²). Waist circumference was measured using a non-stretchable measuring tape at the midpoint between the lower rib margin and the iliac crest. Blood pressure was measured after participants had been seated at rest for at least 15 minutes. Two consecutive readings were obtained using an Omron automated sphygmomanometer. The mean of the two measurements was recorded for analysis.

### 2.3 Biochemical measurements

After overnight fasting, venous blood samples were collected for the assessment of biochemical parameters, including FBG, serum lipid profile, serum creatinine (SCr), and uric acid (UA). The same laboratory methods were applied in both the cross-sectional and cohort analyses, and all measurements were performed in the same hospital clinical laboratory. FBG was measured using an enzymatic method. Serum lipid parameters and UA were determined using enzymatic colorimetric assays, while SCr was assessed using an enzymatic method. All analyses were conducted on a Roche automated biochemical analyzer in accordance with the manufacturer’s instructions and standard laboratory procedures.

### 2.4 Definition of relevant indicators

IR was assessed using the METS-IR (Metabolic Score for Insulin Resistance), calculated as


$$METS-IR=ln[2\times FPG+TG]\times \frac{BMI}{ln(HDL-C)}$$

(17). FPG refers to fasting plasma glucose (mg/dL), TG to triglycerides (mg/dL), and HDL-C to high-density lipoprotein cholesterol (mg/dL). The cardiovascular–kidney–metabolic (CKM) syndrome staging framework, proposed by Chiadi E. Ndumele and colleagues in an American Heart Association scientific statement (18), delineates the pathophysiologic progression across the CKM continuum and the stepwise accumulation of absolute atherosclerotic cardiovascular disease (CVD) risk into five levels (Stages 0–4): Stage 0 denotes absence of CKM risk factors (normal anthropometrics, glycemia, blood pressure, and lipids); Stage 1 captures overweight/obesity or adipose tissue dysfunction (e.g., central adiposity, prediabetes) without additional metabolic risk factors or chronic kidney disease (CKD); Stage 2 comprises one or more metabolic risk factors (e.g., hypertriglyceridemia, hypertension, diabetes, or metabolic syndrome) or CKD at moderate–high risk per KDIGO; Stage 3 reflects subclinical cardiovascular disease or a risk-equivalent state (markedly elevated 10-year predicted CVD risk or very-high-risk CKD); and Stage 4 indicates established clinical CVD.

### 2.5 Definition of diseases

MAFLD was defined as the presence of hepatic steatosis detected by abdominal ultrasonography, together with at least one of the following criteria (19): (1) BMI ≥23 kg/m² or waist circumference (WC) >90 cm in males or >80 cm in females; (2) fasting plasma glucose >5.6 mmol/L (100 mg/dL), or 2-hour post-load glucose ≥7.8 mmol/L (≥140 mg/dL), or HbA1c ≥5.7% (39 mmol/mol), or a diagnosis of type 2 diabetes mellitus, or receipt of treatment for type 2 diabetes; (3) blood pressure ≥130/85 mmHg or use of antihypertensive medication; (4) HDL-C ≤1.0 mmol/L (40 mg/dL) in males and ≤1.3 mmol/L (50 mg/dL) in females, or receipt of lipid-lowering therapy; and (5) triglycerides (TG) >1.70 mmol/L (150 mg/dL) or receipt of lipid-lowering therapy. Kidney stones were defined based on abdominal ultrasonography findings and/or documented diagnoses recorded in the hospital electronic medical record system.

### 2.6 Statistical analysis

All statistical analyses were conducted using RStudio 4.2.1. Normally distributed variables were summarized as mean ± standard deviation (SD), non-normally distributed variables were expressed as median with interquartile range (IQR), whereas categorical variables were presented as frequencies and percentages. Group differences were evaluated by Student’s t test for normally distributed data, by the Kruskal–Wallis test for non-normally distributed data, and by the Chi-square test for categorical data.

In the cross-sectional study, univariate and multivariate binary logistic regression analyses were performed to identify factors associated with the occurrence of kidney stones, and odds ratios (ORs) with 95% confidence intervals (CIs) were reported. In the cohort study analysis, the incidence of kidney stones was expressed per 1,000 person-years. Cumulative incidence was further evaluated using Kaplan–Meier survival curves and the log-rank test. Cox proportional hazards regression was employed to estimate hazard ratios (HRs) and 95% confidence intervals (CIs) of MASLD in relation to kidney stones risk. Two Cox regression models were constructed. Model 1 was unadjusted; Model 2 was adjusted for BMI, sex, age, SBP, DBP, exercise frequency, smoking status, alcohol drinking frequency, and antihypertensive medication use. Subgroup analyses were simultaneously performed according to age, sex, BMI, and CKM stage.

Mediation analyses were performed using the mediation package in R. The potential mediators included METS-IR, white blood cell count (WBC), and estimated glomerular filtration rate (eGFR). A nonparametric bootstrap approach with 1,000 resamples was applied to estimate the average causal mediation effect (ACME), average direct effect (ADE), total effect, and the proportion mediated. All models were adjusted for potential confounders, including BMI, sex, age, mean systolic blood pressure (MSBP), mean diastolic blood pressure (MDBP), exercise frequency, smoking status, alcohol drinking frequency, and antihypertensive medication use, fasting blood glucose (FBG), triglycerides (TG), high-density lipoprotein Cholesterol (HDL-C). Statistical significance was defined as a P-value < 0.05.

## 3 Results

### 3.1 Baseline characteristics of the study population

A total of 1875 participants with an average age of 54.32 ± 14.36 years were included in this cross-sectional study, and 57.39% were males. Of these, 449 had MASLD and 259 had kidney stones, accounting for 23.95% and 13.81%, respectively. Compared with males, females were older, had lower BMI, SBP, DBP, and less frequent smoking and alcohol consumption (P< 0.05). Significant sex differences were also observed in fasting glucose, lipid profiles, hypertension, diabetes, MASLD, and the use of antihypertensive medication.

In the cohort study, 1,903 participants were enrolled at baseline. During a median follow-up of 34.62 (28.28, 38.06) months, 94 (4.94%) incident cases of kidney stones were identified. Compared with females, males had higher BMI, DBP, and METS-IR but lower eGFR and HDL-C (P < 0.05). Smoking and alcohol consumption were much more common among males, while MASLD was more prevalent in females. CKM stage distribution also differed significantly between sexes. During follow-up, the incidence of kidney stones was higher in males than in females (P = 0.006). The baseline in formation is presented in **Tables 1**, **2**.

**Table 1:** Baseline characteristics of the study population in cross-sectional research.

Characteristic	Overall	Male	Female	P
	N = 1,875	N = 1,076	N = 799	
Age (year)	54.32 ± 14.36	53.65 ± 14.57	55.22 ± 14.04	0.042
BMI (kg/m^2^)	24.25 ± 3.77	24.67 ± 3.95	23.69 ± 3.43	<0.001
SBP (mmHg)	139.81 ± 24.31	139.32 ± 24.61	140.48 ± 23.89	0.2
DBP (mmHg)	84.39 ± 15.83	85.14 ± 15.75	83.38 ± 15.89	0.02
Smoking, n(%)				<0.001
Current	252.00 (13.44)	247.00 (22.96)	5.00 (0.63)	
Former	175.00 (9.33)	169.00 (15.71)	6.00 (0.75)	
Never	1,448.00 (77.23)	660.00 (61.34)	788.00 (98.62)	
Alcohol consumption, n(%)				<0.001
Current	166.00 (8.85)	163.00 (15.15)	3.00 (0.38)	
Former	163.00 (8.69)	154.00 (14.31)	9.00 (1.13)	
Never	1,546.00 (82.45)	759.00 (70.54)	787.00 (98.50)	
glucose (mmol/l)	6.61 (5.16, 9.67)	6.70 (5.14, 9.94)	6.47 (5.18, 9.17)	0.3
TC (mmol/l)	4.46 (3.80, 5.25)	4.41 (3.68, 5.21)	4.54 (3.93, 5.29)	0.002
TG (mmol/l)	1.37 (0.96, 1.98)	1.39 (0.96, 2.05)	1.34 (0.96, 1.88)	0.15
HDL-C (mmol/l)	1.10 (0.92, 1.32)	1.03 (0.87, 1.24)	1.19 (1.01, 1.43)	<0.001
LDL-C (mmol/l)	2.77 (2.16, 3.44)	2.75 (2.09, 3.43)	2.80 (2.23, 3.45)	0.2
SUA (mmol/l)	343.00 (275.00, 420.00)	378.00 (308.00, 453.00)	301.00 (247.50, 359.00)	<0.001
Hypertension, n(%)	1,278.00 (68.16)	709.00 (65.89)	569.00 (71.21)	0.014
DM, n(%)	589.00 (31.41)	330.00 (30.67)	259.00 (32.42)	0.4
MASLD, n(%)	449.00 (23.95)	270.00 (25.09)	179.00 (22.40)	0.2
Kidney stones, n(%)	259.00 (13.81)	160.00 (14.87)	99.00 (12.39)	0.12
Antihypertensive medication, n(%)	1,094.00 (58.41)	605.00 (56.23)	489.00 (61.36)	0.026

Data are presented as mean ± standard deviation (SD) for normally distributed variables, median (interquartile range, IQR) for non-normally distributed variables, and number (percentage) for categorical variables. Comparisons between male and female participants were conducted using Student’s t test for continuous variables with normal distribution, the Kruskal–Wallis test for skewed variables, and the Chi-square test for categorical variables. BMI, body mass index; SBP, systolic blood pressure; DBP, diastolic blood pressure; TC, total cholesterol; TG, triglycerides; HDL-C, high-density lipoprotein cholesterol; LDL-C, low-density lipoprotein cholesterol; SUA, serum uric acid; DM, diabetes mellitus; MASLD, metabolic dysfunction-associated steatotic liver disease.

**Table 2:** Baseline characteristics of the study population in cohort study.

Characteristic	Overall	Male	Female	P
	N = 1,903	N = 754	N = 1,149	
Age (year)	69.46 ± 7.66	69.40 ± 8.23	69.49 ± 7.26	0.200
BMI (kg/m^2^)	23.65 ± 3.06	23.86 ± 2.91	23.51 ± 3.15	0.004
SBP (mmHg)	132.48 ± 11.23	132.24 ± 10.50	132.65 ± 11.68	0.300
DBP (mmHg)	80.48 ± 6.75	80.91 ± 6.38	80.19 ± 6.97	0.041
Exercise frequency, n(%)				0.200
Daily	1,735.00 (91.17)	678.00 (89.92)	1,057.00 (91.99)	
Occasionally	34.00 (1.79)	18.00 (2.39)	16.00 (1.39)	
Never	134.00 (7.04)	58.00 (7.69)	76.00 (6.61)	
Smoking, n(%)				<0.001
Never	1,786.00 (93.85)	638.00 (84.62)	1,148.00 (99.91)	
Former	40.00 (2.10)	39.00 (5.17)	1.00 (0.09)	
Current	77.00 (4.05)	77.00 (10.21)	0.00 (0.00)	
Alcohol frequency, n(%)				<0.001
Never	1,806.00 (94.90)	660.00 (87.53)	1,146.00 (99.74)	
Former	56.00 (2.94)	53.00 (7.03)	3.00 (0.26)	
Current	41.00 (2.15)	41.00 (5.44)	0.00 (0.00)	
METS-IR	34.43 ± 6.02	35.40 ± 6.03	33.79 ± 5.93	<0.001
FPG (mmol/l)	5.30 ± 1.65	5.29 ± 1.64	5.31 ± 1.65	0.7
eGFR	94.15 (76.84, 114.27)	78.67 (67.74, 92.34)	106.49 (88.43, 124.27)	<0.001
TC (mmol/l)	4.89 (4.25, 5.59)	4.67 (3.89, 5.32)	5.04 (4.41, 5.72)	<0.001
TG (mmol/l)	1.24 (0.92, 1.72)	1.20 (0.89, 1.67)	1.27 (0.95, 1.75)	0.017
LDL-C (mmol/l)	3.12 (2.48, 3.74)	2.99 (2.29, 3.58)	3.19 (2.61, 3.82)	<0.001
HDL-C (mmol/l)	1.35 (1.14, 1.59)	1.23 (1.06, 1.45)	1.42 (1.21, 1.65)	<0.001
hypertension, n(%)	988.00 (51.92)	395.00 (52.39)	593.00 (51.61)	0.7
DM, n(%)	349.00 (18.34)	139.00 (18.44)	210.00 (18.28)	>0.9
MASLD, n(%)	526.00 (27.64)	187.00 (24.80)	339.00 (29.50)	0.025
Antihypertensive medication, n(%)	740.00 (38.89)	293.00 (38.86)	447.00 (38.90)	>0.9
CKM, n(%)				<0.001
0~1	390.00 (20.49)	122.00 (16.18)	268.00 (23.32)	
2	829.00 (43.56)	274.00 (36.34)	555.00 (48.30)	
3~4	684.00 (35.94)	358.00 (47.48)	326.00 (28.37)	
Time (month)	34.62 (28.28, 38.06)	34.58 (27.98, 37.96)	34.65 (28.48, 38.12)	0.300
Status, n(%)	94.00 (4.94)	50.00 (6.63)	44.00 (3.83)	0.006

Data are presented as mean ± standard deviation (SD) for normally distributed variables, median (interquartile range, IQR) for non-normally distributed variables, and number (percentage) for categorical variables. Comparisons between male and female participants were conducted using Student’s t test for continuous variables with normal distribution, the Kruskal–Wallis test for skewed variables, and the Chi-square test for categorical variables. BMI, body mass index; SBP, systolic blood pressure; DBP, diastolic blood pressure; METS-IR, metabolic score for insulin resistance; FPG, fasting plasma glucose; eGFR, estimated glomerular filtration rate; TC, total cholesterol; TG, triglycerides; LDL-C, low-density lipoprotein cholesterol; HDL-C, high-density lipoprotein cholesterol; SUA, serum uric acid; DM, diabetes mellitus; MASLD, metabolic dysfunction-associated steatotic liver disease; CKM, cardiovascular-kidney-metabolic syndrome.

### 3.2 Cross-sectional analysis identifying MASLD as a risk factor for kidney stones

To evaluate whether MASLD is independently associated with kidney stones, univariate and multivariable binary logistic regression analyses were performed (**Table 3**). In the univariate model, participants with MASLD were associated with a higher risk of kidney stones compared with those without MASLD, with an OR of 1.51 (95% CI: 1.128–2.008). After adjustment for potential confounders, participants with MASLD had approximately a 47% increased odds of developing kidney stones compared with those without MASLD (OR = 1.466, 95% CI: 1.059–2.028), indicating that MASLD is an independent risk factor.

**Table 3:** Univariate and multivariable binary logistic regression analysis of the association between MASLD and kidney stones.

	Univariate		Multivariable	
variable	OR(95%CI)	P	OR(95%CI)	P
Sex	0.81(0.617, 1.058)	0.124	0.903(0.645, 1.265)	0.554
Age	1.006(0.996, 1.015)	0.242	1.01(0.999, 1.022)	0.072
BMI	1.039(1.004, 1.074)	0.025	1.02(0.98, 1.061)	0.331
SBP	1.009(1.004, 1.014)	0.001	1.00(0.991, 1.01)	0.930
DBP	1.014(1.006, 1.022)	0.001	1.013(0.999, 1.028)	0.071
Smoking				
Never				
Former	1.044(0.655, 1.602)	0.849	0.702(0.382, 1.291)	0.255
Current	0.802(0.597, 1.089)	0.151	0.673(0.419, 1.081)	0.101
Alcohol consumption				
Never				
Former	1.266(0.804, 1.928)	0.288	1.694(0.839, 3.419)	0.142
Current	0.983(0.703, 1.4)	0.922	1.589(0.881, 2.865)	0.124
UA	1.001(1, 1.002)	0.074	1(0.999, 1.002)	0.448
MASLD	1.51(1.128, 2.008)	0.005	1.466(1.059, 2.028)	0.021
Antihypertensive medication	1.294(0.988, 1.703)	0.063	1.03(0.755, 1.406)	0.852

OR, odds ratio; CI, confidence interval; BMI, body mass index; SBP, systolic blood pressure; DBP, diastolic blood pressure; UA, uric acid.

### 3.3 Risk of kidney stones associated with MASLD: overall and stratified analyses

**Figure 2:**
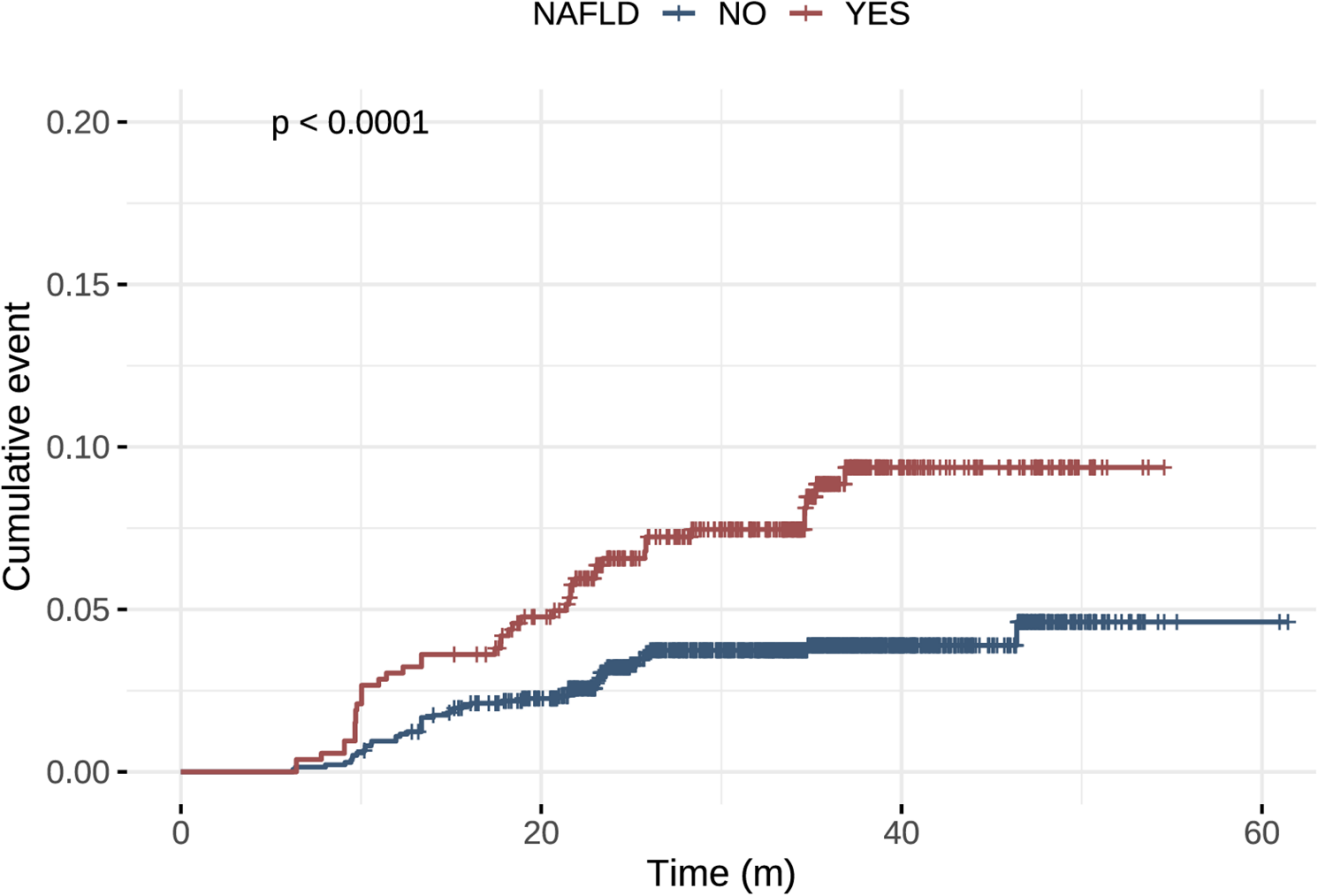
Kaplan–Meier curves for cumulative incidence of kidney stones according to MAFLD status.

The cumulative incidence of kidney stones was significantly elevated in the MASLD group relative to the non- MASLD group, as indicated by the log-rank test (P < 0.001; **Figure 2**).

Over a median follow-up of 34.62 months (IQR: 28.28–38.06), 94 incident kidney stone cases were identified, with an overall incidence rate of 17.6 (95% CI: 14.2–21.6) per 1,000 person-years. In the total population, Compared with participants without MASLD, those with MASLD was significantly associated with an elevated risk of kidney stones, with an HR of 2.22 (95% CI: 1.48–3.32) in the crude model and 2.04 (95% CI: 1.29–3.23) after multivariable adjustment.

In subgroup analyses (**Table 4**), the association between MASLD and incident kidney stones was generally consistent across most strata. When stratified by age, a significant association was observed among participants aged <70 years (HR = 3.09, 95% CI: 1.63–5.86), whereas the association did not reach statistical significance among those aged ≥70 years (HR = 1.48, 95% CI: 0.76–2.89). In sex-stratified analyses, MASLD was significantly associated with an increased risk of kidney stones in male participants (HR = 2.49, 95% CI: 1.13–4.66), while the association was not statistically significant in female participants (HR = 1.66, 95% CI: 0.86–3.22). When stratified by BMI, MASLD was significantly associated with kidney stone risk among participants with BMI ≥24 kg/m² (HR = 2.90, 95% CI: 1.55–5.42), but not among those with BMI <24 kg/m² (HR = 1.26, 95% CI: 0.59–2.71). According to CKM stage, MASLD was significantly associated with kidney stones in participants with CKM stage 2 (HR = 2.69, 95% CI: 1.45–4.98), whereas no significant associations were observed in those with CKM stage 0–1 (HR = 0.62, 95% CI: 0.11–3.35) or stage 3–4 (HR = 2.03, 95% CI: 0.86–4.79).Tests for interaction indicated that the association between MASLD and kidney stones differed significantly by BMI (P for interaction = 0.028).

**Table 4:** The association between MASLD and kidney stones in the total population and subgroups.

Group	Events/Participants	Incidence rate (95% CI)	Model 1			Model 2		
			HR (95%CI)	P	P for interaction	HR (95%CI)	P	P for interaction
Total population	94/1903	17.6 (14.2, 21.6)	2.22(1.48, 3.32)	<0.001		2.04(1.29, 3.23)	0.002	
Age								
<70	48/969	17.9(13.2, 23.8)	2.99(1.70, 5.26)	<0.001	0.155	3.09(1.63, 5.86)	0.001	0.080
≥70	46/934	17.3(12.7, 23.1)	1.65(0.92, 2.98)	0.001		1.48(0.76, 2.89)	0.252	
gender					0.499			0.271
male	50/754	23.9 (17.8, 31.5)	2.61(1.5, 4.56)	0.001		2.49(1.13, 4.66)	0.004	
female	44/1149	13.5 (9.8, 18.2)	1.99(1.1, 3.6)	0.023		1.66(0.86, 3.22)	0.130	
BMI					0.059			0.028
<24	44/1083	14.3 (10.4, 19.3)	1.26(0.62, 2.54)	0.525		1.26(0.59, 2.71)	0.546	
≥24	50/820	22.0 (16.3, 29.0)	3.03(1.69, 5.44)	<0.001		2.90(1.55, 5.42)	0.001	
CKM Stage					0.188			0.266
0~1	12/390	11.4 (5.9, 20.0)	0.65(0.14, 2.97)	0.579		0.62(0.11, 3.35)	0.574	
2	50/829	20.8 (15.5, 27.5)	2.38(1.36, 4.17)	0.002		2.69(1.45, 4.98)	0.002	
3~4	32/684	16.9 (11.6, 23.9)	2.59(1.26, 5.29)	0.009		2.03(0.86, 4.79)	0.104	

Incidence rates are expressed as cases per 1,000 person-years with 95% confidence intervals (CIs). Model 1 was unadjusted. Model 2 was adjusted for body mass index (BMI), gender, age, systolic blood pressure (SBP), diastolic blood pressure (DBP), exercise frequency, smoking status, alcohol consumption frequency, use of antihypertensive medication, fasting blood glucose (FBG), triglycerides (TG), high-density lipoprotein Cholesterol (HDL-C). P for interaction values indicate whether associations differed significantly across subgroups. Due to the limited number of events in participants aged <60 years, hazard ratios (HRs) could not be reliably estimated for this group.

### 3.4 Mediation analysis identifying METS-IR as a key mediator

**Figure 3:**
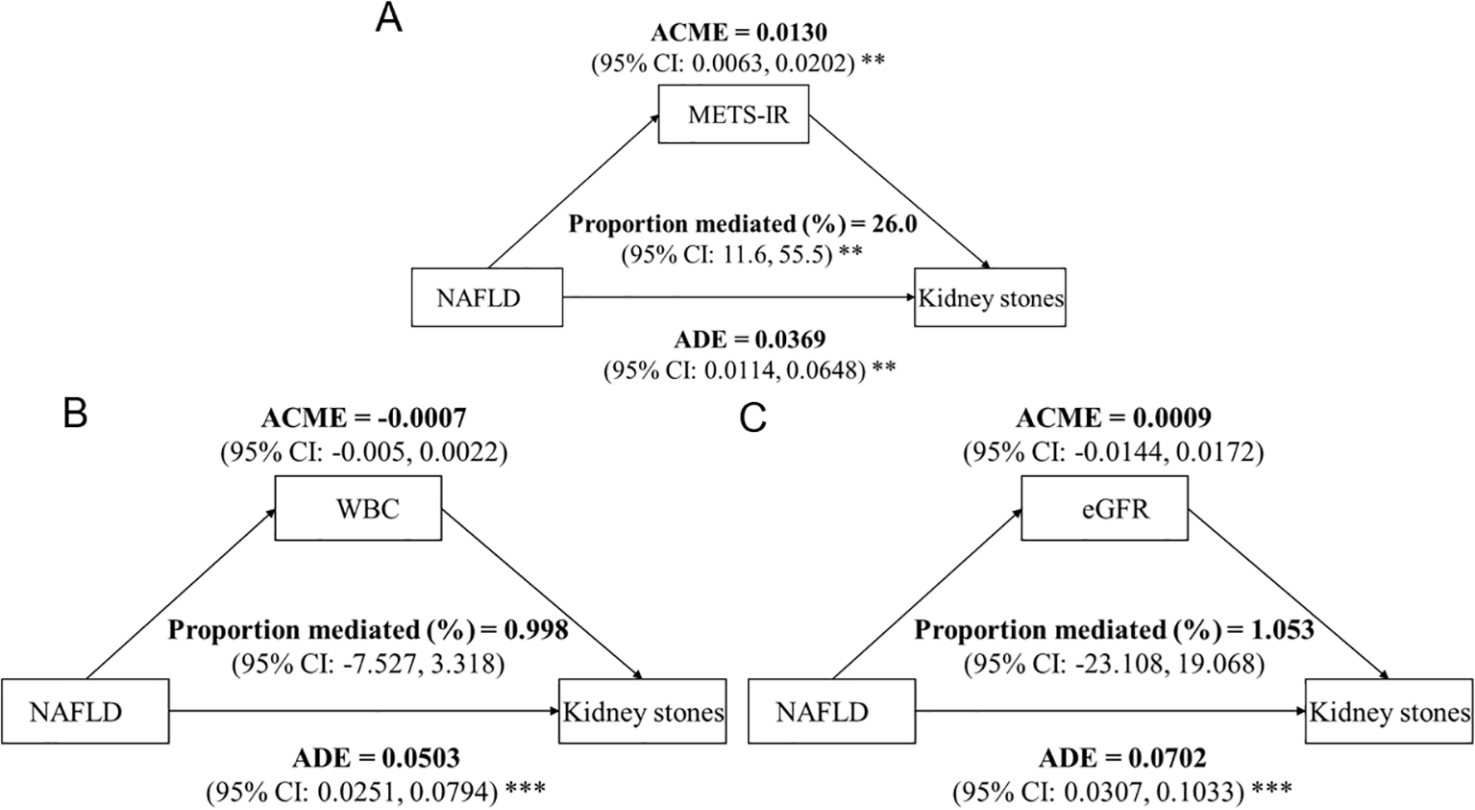
Mediation analysis of the association between NAFLD and kidney stones. The graphs in **(A–C)** represented the mediating role of METS-IR, WBC, eGFR, respectively. The analysis was conducted using the R *mediation* package with 1,000 bootstrap resamples. The metabolic score for insulin resistance (METS-IR), white blood cell count (WBC), and estimated glomerular filtration rate (eGFR) were tested as potential mediators. The average causal mediation effect (ACME), average direct effect (ADE), total effect, and the proportion mediated were estimated, with adjustment for confounders including BMI, sex, age, systolic blood pressure (SBP), diastolic blood pressure (DBP), exercise frequency, smoking status, alcohol drinking frequency, antihypertensive medication use, fasting blood glucose (FBG), triglycerides (TG), high-density lipoprotein cholesterol (HDL-C). Statistical significance is indicated by asterisks: * P < 0.05; ** P < 0.01; *** P < 0.001.

Mediation analyses were conducted to explore potential pathways underlying the association between MASLD and kidney stones (**Figure 3**). As shown in **Figure 3A**, METS-IR demonstrated a significant mediating effect, with an ACME of 0.0130 (95% CI: 0.0063–0.0202), accounting for 26.0% (95% CI: 11.6%–55.5%) of the total association. In contrast, neither WBC nor eGFR showed a significant mediating role. Specifically, the ACME for WBC was −0.0007 (95% CI: −0.0050–0.0022), corresponding to a proportion mediated of 1.0% (95% CI: −7.5%–3.3%) (**Figure 3B**). Similarly, eGFR exhibited a non-significant mediation effect, with an ACME of 0.0009 (95% CI: −0.0144–0.0172) and a proportion mediated of 1.1% (95% CI: −23.1%–19.1%) (**Figure 3C**). Across all models, the ADE of MASLD on kidney stones remained statistically significant, indicating that the association was largely driven by direct pathways rather than mediation through WBC or eGFR.

## 4 Discussion

This study comprehensively evaluated the relationship between MASLD and the risk of kidney stones using both cross-sectional and longitudinal data from hospital- and community-based cohorts. MASLD was identified as an independent risk factor for kidney stones, with consistent associations observed across cross-sectional and prospective analyses. Subgroup analyses further demonstrated that the association was evident only among individuals with metabolic abnormalities, particularly those with elevated BMI or advanced CKM stages. Mediation analyses revealed that METS-IR explained 26.0% of the total effect of MASLD on kidney stones, whereas FBG accounted for a smaller proportion (4.2%), and BMI showed no significant mediation effect. Collectively, these findings suggest that systemic metabolic dysfunction linked to MASLD substantially contributes to the development of kidney stones, underscoring the importance of targeting metabolic disturbances in populations with MASLD.

In this study, MASLD was significantly and independently associated with an elevated risk of kidney stones in the cross-sectional analysis. This finding was further confirmed in longitudinal follow-up data from a community-based cohort, strengthening the validity of the observed association. Previous studies have also suggested that patients with MASLD may face a greater risk of urolithiasis, and this relationship has been documented in different populations and settings. For instance, an analysis of 11,859 adults from the U.S. NHANES database demonstrated a significant association between NAFLD and kidney stones in women (OR = 1.65, 95% CI: 1.17–2.32), although no statistical significance was observed in men (20). A Korean study (n = 1381) reported an even stronger association (OR = 4.99, 95% CI: 3.0–8.2) (14). Similarly, a Chinese community-based study (n = 3719) showed that NAFLD was independently associated with kidney stones (OR = 1.35, 95% CI: 1.01–1.81), and the risk increased proportionally with the severity of hepatic steatosis (21). Most recently, epidemiological and genetic analyses from a Chinese population further confirmed a causal relationship between NAFLD and kidney stone formation (15). However, the majority of these investigations were cross-sectional or based on genetic evidence, limiting causal inference. By integrating both cross-sectional and prospective cohort data within the same regional population, our study provides stronger and more consistent evidence, indicating that the association between MASLD and kidney stones is unlikely to be explained by chance. These results underscore the importance of considering kidney stone risk when evaluating the extrahepatic burden of MASLD.

Subgroup analyses demonstrated that the relationship between MASLD and kidney stones was more evident in metabolically unhealthy individuals, especially among those with elevated BMI or CKM stage 2. This pattern suggests that metabolic disturbances may not only modify but also amplify the lithogenic effect of MASLD. MASLD is increasingly understood as a systemic phenotype of metabolic dysfunction rather than a disease confined to the liver, with accumulating evidence showing its close interplay with renal metabolism. These findings underscore that patients with MASLD—particularly those presenting with metabolic abnormalities—should be considered a high-risk group for kidney stone formation. Integrating metabolic intervention with routine hepatology care may therefore provide a dual benefit, reducing both hepatic progression and the burden of urological complications. MASLD is commonly accompanied by systemic low-grade inflammation and increased oxidative stress (22, 23). Population-based studies have also observed a lower prevalence of kidney stones among individuals with higher serum antioxidant levels (24). Taken together, these observations suggest that MASLD-related inflammatory and oxidative stress states may provide a permissive background for kidney stone formation. In addition, MASLD is characterized by impaired hepatic bile acid synthesis, transport, and enterohepatic circulation, leading to dysregulation of bile acid metabolism (25, 26). Such MASLD-related bile acid disturbances may indirectly affect urinary solute composition through gut–liver–kidney axis mechanisms. Altered bile acid handling can impair fat absorption and reduce intestinal calcium availability, thereby increasing colonic oxalate absorption and urinary oxalate excretion (enteric hyperoxaluria), a recognized risk factor for calcium oxalate stone formation (27, 28). Moreover, experimental and clinical studies suggest that changes in bile acid profiles, particularly elevated secondary bile acids, may influence gut microbiota and renal tubular microenvironments, potentially promoting crystal adhesion, oxidative stress, and crystal retention (29, 30). These mechanisms highlight how metabolic dysfunction serves as a catalyst, intensifying the renal consequences of MASLD.

An intriguing finding was that METS-IR significantly mediated the association between MASLD and kidney stones, explaining 26% of the total effect. To our knowledge, this is the first report of such an observation. METS-IR, a noninvasive surrogate of systemic IR, has been widely applied in large-scale epidemiological studies (17, 31). Prior research has shown that glycemic control mediated the link between MASLD and microvascular complications, highlighting the central role of glucose metabolism in MASLD-related outcomes (32).

MASLD is commonly accompanied by IR (33, 34). Previous studies have shown that IR associated with MASLD is frequently linked to chronic low-grade inflammation and increased oxidative stress, which may contribute to extrahepatic organ dysfunction (35). With regard to the potential involvement of IR in kidney stone formation, existing literature has proposed several plausible mechanisms. First, at the renal level, IR has been associated with altered tubular reabsorption of sodium and calcium, which may increase the risk of hypercalciuria and create a more lithogenic renal microenvironment (36). Second, IR may interfere with renal acid excretion and purine metabolism, leading to lower urinary pH and reduced ammonium excretion, thereby increasing the propensity for uric acid and calcium oxalate stone formation (11, 12). It should be noted, however, that direct mechanistic studies specifically linking MASLD to kidney stone formation remain limited. Therefore, the association between MASLD and kidney stones may, at least in part, be related to the accompanying systemic IR state, although the precise biological pathways warrant further investigation.

One major strength of this study lies in its combined use of cross-sectional and longitudinal designs. The complementary nature of these approaches not only enhanced the consistency and robustness of the findings but also strengthened causal inference. Furthermore, the broad inclusion of participants across different age groups and metabolic profiles increased the representativeness and generalizability of the results. Moreover, the study was based on real-world health examination data, which provided a reliable source of information and allowed the epidemiological characteristics of MASLD and kidney stone disease in the general population to be more accurately reflected. Nonetheless, several limitations should be acknowledged. First, dietary habits, which are well-recognized determinants of both MASLD and kidney stone formation (37, 38), were not assessed, and therefore, future studies should incorporate this factor into study design. Second, the diagnosis of MASLD relied on ultrasonography. Although this method is highly feasible for large-scale populations, it lacks quantitative assessment of hepatic steatosis and precludes investigation of dose–response relationships, which may have underestimated the true association between MASLD and kidney stones. Finally, the study was conducted in a single center and limited to a population from a city in western China; consequently, caution is required when extrapolating these findings to other regions or populations.

## 5 Conclusion

Our study suggests that MASLD is associated with a higher risk of kidney stone disease, with broadly consistent findings observed in both cross-sectional and longitudinal cohort analyses. The findings not only underscore MASLD as an important risk factor for kidney stones but also highlight the pivotal role of metabolic dysfunction in mediating this link, thereby revealing the complex pathophysiological interplay between metabolic disorders and urolithiasis. The identification of a likely causal connection between MASLD and kidney stone formation further suggests opportunities for early intervention, emphasizing the importance of targeted prevention and clinical management strategies, particularly among individuals with concomitant metabolic abnormalities.

## Data Availability

The raw data supporting the conclusions of this article will be made available by the authors, without undue reservation.
